# One-stage vs two-stage bilateral THA in Lombardy: a cost-effectiveness analysis

**DOI:** 10.1186/s12962-023-00418-y

**Published:** 2023-01-16

**Authors:** Pierluigi Pironti, Andrea Ambrosanio, Valeria Vismara, Marco Viganò, Eugenia Bucci, Paolo Sirtori, Giuseppe M Peretti, Laura Mangiavini

**Affiliations:** 1 Residency Program in Orthopedics and Traumatology, University of Milan, Milan, Italy; 2 IRCCS Istituto Ortopedico Galeazzi, Via R. Galeazzi 4, 20161 Milan, Italy; 3 Department of Biomedical Sciences for Health, University of Milan, Milan, Italy

**Keywords:** Bilateral total hip arthroplasty, Anterior approach, Cost-effectiveness

## Abstract

**Background:**

Total hip arthroplasty (THA) is the most common treatment for primary and secondary end-stage hip osteoarthritis (OA). Almost 20% of all patients undergoing primary THA suffer from bilateral hip OA and, consequently, will need a contralateral procedure to be performed in the following years. The aim of this study is to evaluate the cost-effectiveness and the reliability of one-stage bilateral THA (1-BTHA) compared to two-stage bilateral THA (2-BTHA), in low-risk patients, performed with anterior minimally invasive surgery (AMIS).

**Methods:**

Single patient’s costs were obtained by dividing the annual costs report by the number of hospitalizations, considering the diagnosis related group (DRG) of the two procedures. Then, 16 patients undergoing 1-BTHA and 8 undergoing 2-BTHA were examined. Hemoglobin (Hb) values before surgery and before discharge, transfusion rate and the occurrence of post-operative complications were observed.

**Results:**

Procedural costs were divided in different subgroups: pre-hospitalization, operating room, hospital stay, post-operative follow-up and other costs. 1-BTHA total costs amount to 5.754,82€, while performing 2-BTHA costs 7.624,32€. However, considering DRG reimbursement, the hospital’s profit margin following 1-BTHA is lower than that following 2-BTHA (6.346,18€ versus 9.261,68€). Surgical time was found not to be significantly different between 1-BTHA and 2-BTHA (141,13 ± 26,1 min vs 164,8 ± 44,3 min; p = 0,111). The two groups showed a statistically significant difference in Hb decrease (4,8 ± 1,3 g/dl vs 3,3 ± 0,9; p = 0,001), despite no variances in transfusion rate. No further complications were observed in either group.

**Conclusions:**

This study demonstrates how, in carefully selected patients, 1-BTHA performed with AMIS is a cost-effective and safe technique compared to 2-BTHA, resulting in a shorter OR time, LOS and lower overall costs.

**Level of evidence:**

III

## Introduction

Total hip arthroplasty (THA) represents the most common treatment for end-stage hip osteoarthritis (OA), with a high patient satisfaction and a significant improvement in the quality of life [1, 2]. Hip OA has an estimated prevalence of 7.7% in adult population over 65 years old, and 4.4% in the adult over 55 years old [3]. Primary hip OA represents the majority of total disease burden [4]. Several clinical conditions can influence the development of secondary hip OA, such as avascular necrosis, rheumatoid arthritis, ankylosing spondylitis, and congenital dysplasia [1, 5]. It has been observed that almost 20% of all patients who underwent primary THA suffer from bilateral hip OA and, consequently, will need a contralateral procedure to be performed in the following years [3, 6]. Moreover, several studies have suggested the safety and reliability of one-stage bilateral THA (1-BTHA) in low-risk patients [7], showing a reduction of the length of stay (LOS), a shorter anesthesia and surgery time, a faster rehabilitation and a better cost-effectiveness [7, 8]. As the Italian national healthcare service shifted to the Diagnosis-related group (DRG) payment system, patients with similar clinical conditions are grouped, and the hospitals are reimbursed the established amount per admission. Moreover, the Italian health service is further subdivided into Regional Health Service (RHS). Each region, therefore, has the possibility to independently choose the reimbursement amount corresponding to each DRG [9, 10].

Thus, the aim of this study is to evaluate the cost-effectiveness of 1-BTHA compared to two-stage bilateral THA (2-BTHA), taking into account the reimbursement by the public RHS in Lombardy, and the reliability of each procedure.

## Methods

This retrospective, single center study was conducted at IRCCS Istituto Ortopedico Galeazzi, Milan, Italy. Data were collected from patients inserted in the Institutional Registry (H&K Datareg Register).

We included patients between 18 and 85 years, both male and female, with intact cognitive capacity and no serious chronic diseases according to the American Society of Anesthesiologists (ASA ≤ 2), affected by either primary or secondary symptomatic hip OA, in the period between January 2018 and February 2021. Our exclusion criteria were: age > 85 years, patients with low autonomy with activities of daily living, patients affected by neurologic, orthopedic and muscular comorbidities limiting functionality, patients with severe chronic diseases (ASA > 2), patients undergoing revision arthroplasty, as well as major psychiatric disorders (such as schizophrenia, bipolar disorder, depression, uncontrolled anxiety, obsessive-compulsive disorder), pregnancy, severe hip malformations and anti-coagulant medication use. All the patients underwent physical examination prior to the surgery. Plain radiographs were performed to confirm the diagnosis and to assess OA grade.

Data included information about total hospital costs, operating room (OR) time, surgery time and LOS. Cost analysis was performed by our administrative offices. Single patients’ costs were obtained by dividing the annual costs report by the number of hospitalizations, considering the DRG code of the two procedures analyzed according to our patients’ privacy policy. Additionally, in the considered time range, patients who underwent either 1-BTHA or 2-BTHA were examined to compare the reliability of both surgical techniques. Respectively, 16 and 8 patients were identified. Standard surgical procedures were applied in both groups. Hemoglobin (Hb) values before surgery and before discharge, and transfusion rate were observed to compare the blood loss between the two groups. Transfusions were administered either when Hb levels were < 8 g/dl or when the patients were symptomatic for anemia. Furthermore, the occurrence of post-operative complications such as infections, dislocations, pulmonary embolism and thrombosis was evaluated.

### Peri-operative management

**Fig. 1:**
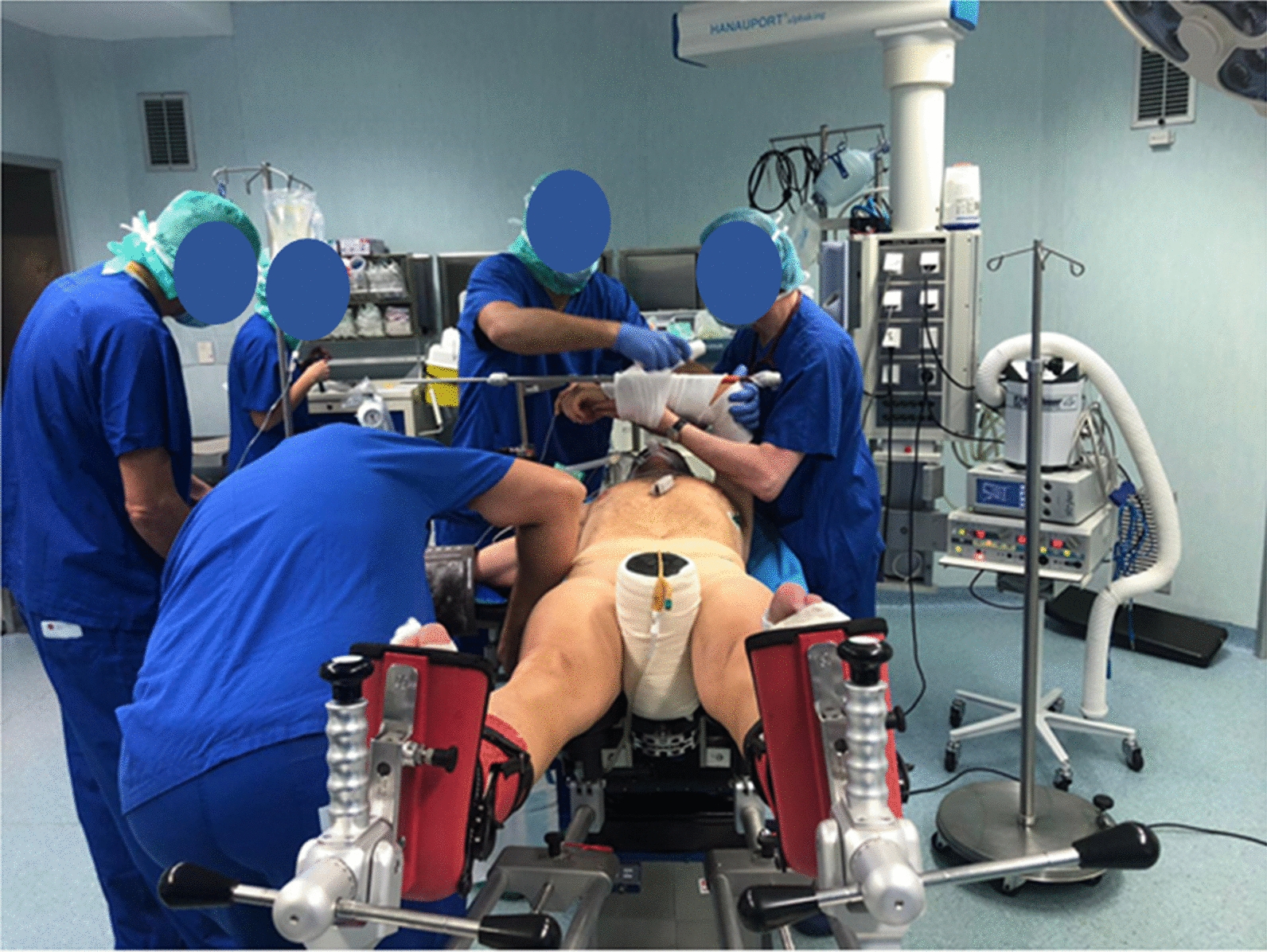
1-BTHA patient positioning using the mobile leg positioner

All the patients followed the fast track (FT) program. The surgical procedure was performed the same day of hospitalization, except for patients requiring blood products on hand for the procedure and patients scheduled as the first procedure of the day, who were hospitalized the previous day. The surgery was performed in regional anesthesia by the same surgical team for each patient. THA was performed using anterior minimally invasive surgery (AMIS), consisting of an anterior surgical approach combined with the employment of a mobile leg positioner, which allows better leg control (Fig. 1). Either SMS or AMIStem femoral component combined with Versafit or Mpact cup with highly cross-linked polyethylene (HXLPE) or Ceramic liner were employed (Medacta, Castel San Pietro, Switzerland); ceramic femoral head was implanted in all prostheses. The surgical approach was performed with an approximately 7 cm incision, starting about 2 cm lateral and 1 cm distal to the anterior superior iliac spine and proceeding distally toward the Gerdy tubercle. The prostheses insertion was performed following the manufacturer’s instructions for use. Two 500 mg/5 ml tranexamic acid TXA vials were administered systemically, and one vial locally if there was no contraindication to reduce post-surgical blood loss [11]. A local infiltration analgesia (LIA) was also performed to prevent excessive post-operative local pain, using one vial of lidocaine 10 mg/ml. No suction drainage was placed, and all the patients received pre-operative antibiotic prophylaxis (cefazolin 2 g iv 30–60 min before the surgery) and anti-thrombotic prophylaxis with enoxaparin sodium 4000 IU for 35 days after surgery.

### Costs analysis

All costs were analysed retrospectively using Excel, Qlickview and Giada software. 2-BTHA consists of two unilateral THA (UTHA) performed in two different hospitalizations, thus the 2-BTHA costs were calculated as double the cost of one UTHA. The costs were subdivided in four main items plus one for other costs. Specifically, we considered pre-hospitalization, operating room, hospital stay, post-operative follow-up and indirect costs. Concerning pre-hospitalization, we considered blood tests, chest X-rays, pelvis X-rays, hip X-rays (twice when 1-BTHA was performed), SARS-COV-2 swab performed 48 h before the surgery (since 2020), anesthesiologic interview and orthopedic visit. OR costs included the prosthesis, materials, intensive care (when applicable) and surgical staff including surgeons, anesthesiologists, nurses, healthcare professionals as well as employees designated to sterilization. Regarding hospital stay costs, we considered the mean LOS, the medical and nursing staff, post-operative blood tests as well as post-operative X-rays, drugs, physiotherapy, services and materials. Post-operative follow-up costs include the first clinical check-up and administrative staff. Indirect costs include all the annual expenses surrounding maintenance, equipment rental, special waste disposal and purchase of health services. Moreover, VAT costs must be considered in our analysis, which correspond to 10% for the operative room material, 4% for prosthesis, 22% for the sterilization material and disposable surgical drapes. For other services (cafeteria, laundry, cleaning, rental and maintenance) VAT corresponds to 22%.

### Statistical analysis

Statistical analysis was performed using R Software v4.0.3 (R Core Team, Wien, Austria). Numerical variables were tested by Shapiro-Wilk test to assess normal distribution. T test or Mann-Whitney U test were used according to the results of normality test to evaluate between groups differences. Fisher’s exact test (or, if not applicable, Chi-square test) was used to test differences between groups in regard to categorical variables. Data are reported as mean ± standard deviation. P values < 0.05 were considered statistically significant.

## Results

**Fig. 2:**
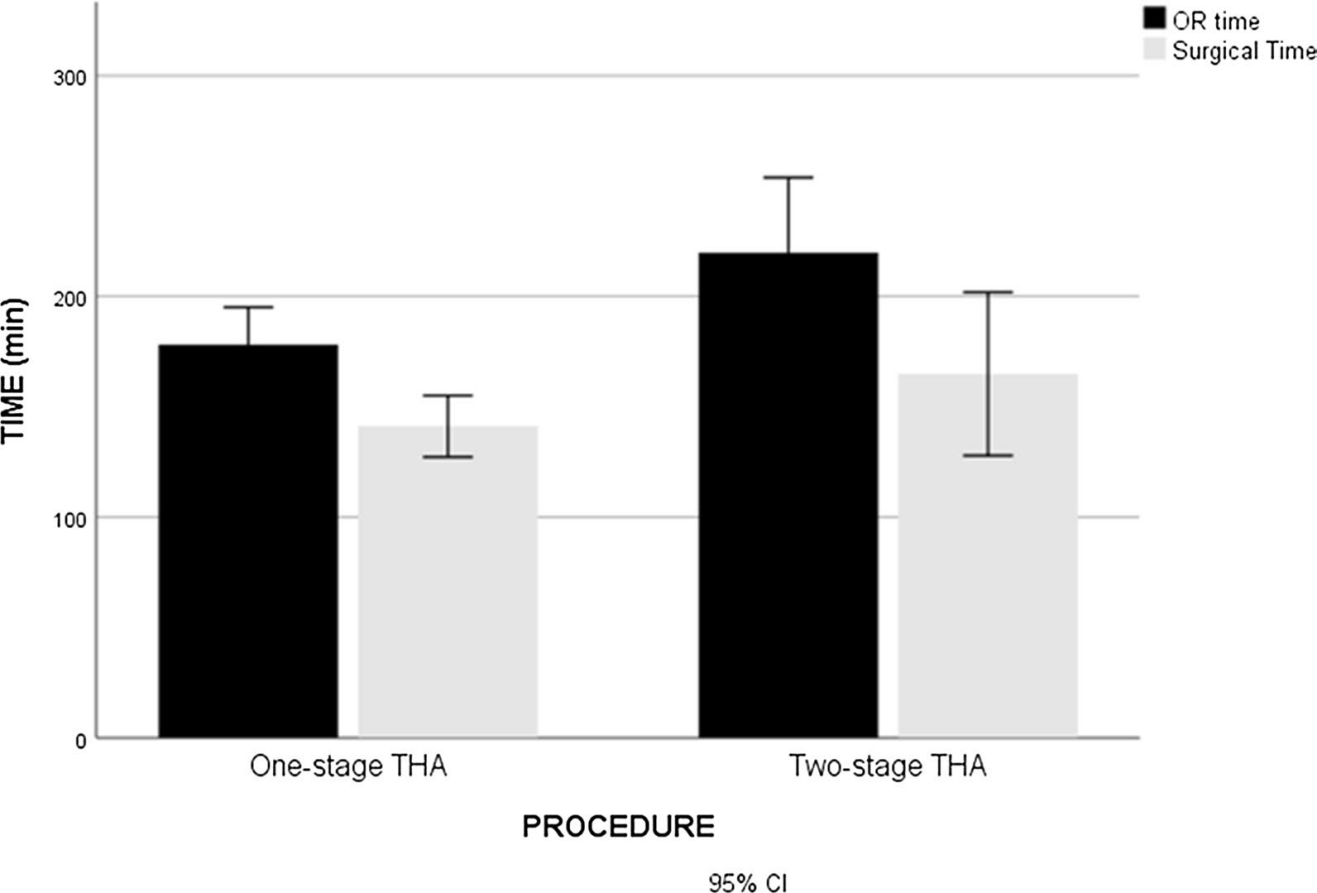
OR time and surgical time of 1-BTHA and 2-BTHA

For the present study, 16 surgeries for 1-BTHA and 16 surgeries for 2-BTHA have been evaluated. Thus, 16 patients underwent 1-BTHA, while 8 patients underwent 2-BTHA. In the first group, out of 16 patients 12 were males, whereas in the second group out of 8 patients only one was male. No significant differences were found among groups when mean age was compared. Primary hip OA was the main indication for treatment, even though one patient was diagnosed with Perthes disease, and two patients had a bilateral osteonecrosis, in the 1-BTHA group. Blood analysis before surgery revealed that a significant difference was found between the two groups: patients that were planned for 1-BTHA started from a higher hemoglobin (Hb) level compared to two-stage technique (mean Hb value were 14,8 g/dl vs 13,5 g/dl). When considering surgery time, despite the significant difference found when comparing 1-BTHA with unilateral THA (141,13 ± 26,1 min vs 82,4 ± 24,5 min; p < 0,0001), this difference is no longer significant when 2-BTHA is considered (141,13 ± 26,1 min vs 164,8 ± 44,3; p = 0,111) (Fig. 2). Patients who underwent one-stage surgery on average lost 4,8 ± 1,3 g/dl (32,22%) Hb points compared to their pre-surgical value, while when performing 2-BTHA the average loss amounted to 3,3 ± 0,9 (24,89%) for each surgery. This difference is statistically significant (p = 0,001). Blood transfusion was needed only in one case for each group. No further complications were developed in either group.

Procedural costs were divided in different subgroups: pre-hospitalization, operating room, hospital stay, post-operative follow up and other costs. The OR costs in 1-BTHA and UTHA had the greatest influence in the final outcome (67% in 1-BTHA vs 58% in UTHA), followed by those related to the hospital stay (24% in 1-BTHA vs 30% in UTHA) (Table 1). 2-BTHA consists of two UTHA performed in two different hospitalizations. Therefore, to obtain the real costs of 2-BTHA total costs of UTHA must be duplicated.

**Table 1:** Procedural costs average either for one stage bilateral THA (1-BTHA) and unilateral THA (UTHA)

	Pre-hospitalization	OR	Hospitalization	Others	Follow-up	Total
1-BTHA	3%	67%	24%	4%	2%	100%
UTHA	4%	58%	30%	6%	2%	100%

Pre-hospitalization costs in the 1-BTHA group amounted to 193,94€ compared to 351,88€ for 2-BTHA. OR costs in the 1-BTHA group amounted to 3.740,97€, which costs less than performing 2-BTHA (4.279,5€). Contributing factors to this outcome are operating room time (mean value being 178,1 ± 31,8 min for 1-BTHA vs 219,8 ± 40,8 min in case of 2-BTHA; p = 0,012) (Fig. 2), material costs (297,46€ vs 546,92€) and OR staff costs (919,29€ vs 1.184,14€). Moreover, the present study showed how LOS was considerably lower in patients who underwent 1-BTHA (5,6 ± 2,1 days vs 8,5 ± 1,9 days; p = 0,003) (Table 2). Accordingly, hospitalization costs were substantially lower compared to patients undergoing 2-BTHA (1.348,51€ vs 2.189,7€). Post-operative follow-up amounted to 107,89€ in 1-BTHA and 181,78€ in 2-BTHA, and indirect costs were respectively 215,32€ and 430,64€. All the details about tariffs are summarized in Table 3.

**Table 2:** Length of stay (LOS), surgical time and operating room (OR) time of one-stage bilateral THA (1-BTHA) and two-stage bilateral THA (2-BTHA)

	LOS (days)	Surgical time (min)	OR time (min)
1-BTHA	5.6 ± 2.1	141.13 ± 26.1	178.1 ± 31.8
2-BTHA	8.5 ± 1.9	164.8 ± 44.3	219.8 ± 40.8
P-value	0.003	0.111	0.012

**Table 3:** Procedural costs and profit margin of one-stage bilateral THA (1-BTHA), two-stage bilateral THA (2-BTHA) and unilateral THA (UTHA)

	1-BTHA	2-BTHA	UTHA
Pre-hospitalization	193,94€	351,88€	175,94€
OR costs	3.740,97€	4.279,5€	2.139,75€
Prosthesis	2.500€	2.500€	1.250€
Materials	297,46€	546,92€	273,46€
OR staff costs	919,29€	1.184,14€	592,07€
Post-operative intensive care	24,22€	48,44€	24,22€
Total hospitalization costs	1.348,51€	2.189,7€	1.094,85€
Blood tests	204,88€	409,76€	204,88€
Blood availability	53,76€	107,52€	53,76€
Hospital stay	899,47€	1.383,42€	691,71€
Physiotherapy	56€	85€	42,5€
Services and utilities	134,4€	204€	102€
Others	215,32€	430,64€	215,32€
Administration	50,32€	100,64€	50,32€
Facilities	165€	330€	165€
Follow-up	107,89€	181,78€	90,89€
VAT	148,19€	190,82€	95,41€
Total cost (including VAT)	5.754,82€	7.624,32€	3.812,16€
DRG reimbursement	12.101€	16.886€	8.443€
Profit margin	6.346,18€	9.261,68€	4.630,84€

## Discussion

The Italian national healthcare system (NHS) is a public service, regionally decentralized and financed by tax revenue, which provides a comprehensive coverage to all Italian citizens and, since 2002, to all foreign citizens with legal residence [10]. In the past decades, the financing method adopted by the NHS shifted from a delivered product reimbursement to predetermined charges for performance, classified by DRG [10, 12]. The DRG charges are intended to cover most hospital costs, including administrative costs and outlays with the exclusion of capital costs [13]. The national DRG tariffs are set equal for all types of providers. However, regional variations are allowed [13, 14]. To date, almost all regions apply different tariffs to different providers, classified according to various criteria which take into account organizational features and services provided [13]. The current reimbursement for THA in Lombardy amounts to 8.443€, while for 1-BTHA amounts to 12.101€. It must be noticed that, since 1-BTHA involves the implant of two prostheses during the same hospitalization period, some costs, such as post-operative intensive care (when necessary), blood tests, blood availability, facilities and administration costs, are similar to those for UTHA (Table 3). Costs related to OR materials and staff are higher in 1-BTHA because of the longer surgical time and the employment of additional materials such as sterile disposable cloths (Table 3). Concerning hospitalization, a greater amount of total costs in 1-BTHA can be observed due to the slightly higher LOS (5,6 ± 2,1 days vs 4,25 ± 1,5; p = 0,05). Considering that total costs of unilateral THA must be duplicated to obtain the real costs of 2-BTHA, the total expense for 1-BTHA is considerably lower (Table 3). In fact, while in the first case total costs amount to 5.754,82€, performing UTHA costs 3.812,16€. This amount has to be duplicated in order to obtain the real value of 2-BTHA, which is substantially superior to the one obtained in the first group we analyzed (7.624,32€) (Table 3). All in all, what emerged is that performing a 1-BTHA costs less than performing two surgeries in different times. However, considering the current reimbursement of 12.101€ for 1-BTHA, the hospital profit margin is lower than the reimbursement that could be obtained performing two procedures separately (6.346,18€ instead of 9.261,68€) (Table 3).

1-BTHA could provide fewer cardiopulmonary complications, incidence of deep venous thrombosis (DVT), and no significant difference in blood loss, likely to be related to the shorter anesthesia and surgical time [1, 15]. Furthermore, 1-BTHA provides a single hospitalization, risks associated with only one anesthetic, a shorter rehabilitation time and a higher patient satisfaction [6]. For instance, Taheriazam et al. stated that 1-BTHA can be employed without any increase in complication rate, showing clinical and functional outcomes comparable to 2-BTHA and a substantially shorter LOS [3]. Huang et al. observed lower incidence of DVT, pulmonary embolism and respiratory complication rate in 1-BTHA compared to 2-BTHA, stating that the first one is a safer procedure in selected patients [8]. Furthermore, Kutzner et al. conducted a study demonstrating that 1-BTHA is safe to perform, showing a low complication rate and good short-term functional outcomes [16]. Recently, the direct anterior approach for THA has become widespread in clinical orthopedic practice, demonstrating satisfying outcomes. In fact, it seems to be a feasible option for one-stage bilateral THA because of its minimally invasive characteristics [15]. Tamaki et al. investigated the perioperative blood loss and complications of 1-BTHA performed with the direct anterior approach, showing a low rate of systemic complications [15]. Also, Petridis and Nolde established the reliability of the aforementioned approach by assessing the clinical outcomes and complications [7].

Our experience confirmed that 1-BTHA using AMIS is a reliable technique. Although 1-BTHA group showed a statistically significant difference in Hb decrease, there were no differences in transfusion rate between the two groups. Moreover, it must be considered that, despite the more extensive surgical exposure of 1-BTHA given the bilateral incision the mean blood loss was still acceptable. Additionally, our cost analysis showed that 1-BTHA, rather than 2-BTHA, could be advantageous in term of cost-effectiveness when performed in selected patients, as stated by other authors [3, 6, 14]. Considering the shorter total OR time and LOS, performing 1-BTHA could lead to an improvement in OR availability and decrease in wait list time [17].

### Limits

Despite the encouraging results, several limitations must be addressed. Firstly, a larger sample would have been preferred to avoid selection biases. The small sample could be attributed to a limited number of patients with the surgical indication of bilateral THA in the time range taken into consideration. Indeed, the observation period was limited because we considered only patients treated with AMIS, which was introduced in our clinical routine from 2018. Furthermore, since 1-BTHA is less affordable for the hospital because of the lower DRG reimbursement, we did not have the possibility to operate a larger number of patients using this technique. In respect of our patients’ privacy policy, the administrative office which conducted the cost analysis did not have the access to the patients’ personal details. Thus, considering 2-BTHA is performed in two different hospitalizations, its costs were considered to be twice the amount of UTHA. The follow-up was limited to the hospitalization period because only perioperative complications were considered. Moreover, no other surgical approach except for AMIS was evaluated in the present study.

## Conclusions

In conclusion, this study demonstrated that 1-BTHA performed with AMIS is a cost-effective technique compared to 2-BTHA, demonstrating a shorter OR time, LOS and lower overall costs. Moreover, it confirmed that 1-BTHA is safe to perform in selected patients, with no perioperative complications, and a tolerable blood loss in comparison to 2-BTHA. However, considering the DRG reimbursement in Lombardy, the hospitals are not incentivized to promote this procedure. Thus, an increase of the regional reimbursement could lead to several benefits for the entire RHS and, most importantly, for the patients.

## Data Availability

All data generated or analyzed during this study are included in this published article.

## Abbreviations

THA: Total hip arthroplasty
OA: Osteoarthritis
1-BTHA: One-stage bilateral THA
LOS: Length of stay
DRG: Diagnosis related group
RHS: Regional health service
2-BTHA: Two-stage bilateral THA
ASA: American Society of Anesthesiologists
OR: Operating room
Hb: Hemoglobin
FT: Fast track
AMIS: Anterior minimally invasive surgery
HXLPE: Highly cross-linked polyethylene
TXA: Tranexamic acid
LIA: Local infiltration analgesia
UTHA: Unilateral total hip arthroplasty
NHS: National healthcare system
DVT: Deep venous thrombosis
