# Correction: One-stage vs two-stage bilateral THA in Lombardy: a cost-effectiveness analysis

**DOI:** 10.1186/s12962-023-00419-x

**Published:** 2023-01-25

**Authors:** Pierluigi Pironti, Andrea Ambrosanio, Valeria Vismara, Marco Viganò, Eugenia Bucci, Paolo Sirtori, Giuseppe M. Peretti, Laura Mangiavini

**Affiliations:** 1 Residency Program in Orthopedics and Traumatology, University of Milan, Milan, Italy; 2 IRCCS Istituto Ortopedico Galeazzi, Via R. Galeazzi 4, 20161 Milan, Italy; 3 Department of Biomedical Sciences for Health, University of Milan, Milan, Italy

**Correction: Cost Effectiveness and Resource Allocation (2023) 21:3** **https://doi.org/10.1186/s12962-023-00418-y**

Following publication of the original article [1], the authors notified us that the following funding information had been omitted from the Funding declaration: This work was supported and funded by the Italian Ministry of Health ('Ricerca Corrente').

The published article has now been updated with this funding information. The authors thank you for reading and apologize for any inconvenience caused.
